# Improvements in habitability and housing satisfaction after dwelling regeneration in social housing complexes. The RUCAS study^☆^

**DOI:** 10.1016/j.socscimed.2024.117090

**Published:** 2024-08

**Authors:** F. González, F. Baeza, R. Valdebenito, B.N. Sánchez, A. Diez-Roux, A. Vives

**Affiliations:** a Department of Public Health, School of Medicine, Pontificia Universidad Católica de Chile, Chile; b Institute of Geography, Pontificia Universidad Católica de Chile, Chile; c Department of Epidemiology and Biostatistics, Dornsife School of Public Health, Drexel University, Philadelphia, USA; d Centre for Sustainable Urban Development, CEDEUS, Chile

**Keywords:** Social housing, Residential satisfaction, Housing satisfaction, Natural experiment, Dwelling renovation, Urban regeneration, RUCAS project, Central Chile, Low-income neighborhoods

## Abstract

Housing is a pressing problem worldwide and a key determinant of health and wellbeing. The right to adequate housing, as a pillar of the right to an adequate standard of living, means more than a roof to live under. Adequate means the dwelling must fulfill material functions and psychosocial functions, thus contributing to dwellers health and wellbeing. Social housing policies aim to fulfill the right to housing, but frequently fail in fulfilling the right to it being adequate.

This study capitalizes on the implementation of a national urban regeneration program in two social housing villas in central Chile (one in Santiago, in the central valley, the other in Viña del Mar, a coastal city) to run a natural experiment assessing the impact of dwelling renovation on several dimensions of perceived habitability and housing satisfaction among the -mostly female-household homemakers.

We use 5 waves of survey data collected with a step-wedge design to estimate the association between a time-varying exposure status (the intervention) and 7 binary outcomes for habitability and 5 for housing dissatisfaction, including overall housing satisfaction. We use Poisson regression models with robust variance and a random intercept at the respondent level. At baseline, reports of poor habitability and dissatisfaction across all features were markedly high, the highest levels of dissatisfaction being with acoustic insulation and dwelling size in both villas, and with indoor temperature in Santiago. The intervention resulted in statistically significant and markedly large improvements in reported habitability and dissatisfaction relative to those housing components targeted by the intervention, as well as with overall dwelling satisfaction in both study cases.

Implications are, first, that the policy response to quantitative housing deficits must not overlook housing quality; second, that housing renovation appears as a promising intervention for qualitative housing crises; third, that while improvements in habitability and satisfaction are specific to the interventions in place, overall housing satisfaction can improve in more limited, tailored, dwelling renovation interventions. Social housing renovation in Latin America appears as a promising intervention to improve quality of life among the urban poor dwellers and reduce inequalities in health related to housing conditions.

## Highlights

• Study of reported habitability and housing satisfaction among urban poor homemakers.
• First longitudinal assessment of formal social housing renovation in Latin America.
• Housing renovation can positively impact quality of life and wellbeing.
• Results stress the need to addressing the qualitative housing crisis.
• General satisfaction improved in all cases, despite different interventions.

## 1 Introduction

### 1.1 Importance of housing to health, wellbeing and quality of life

Housing is a key determinant of health and wellbeing. Several dimensions of housing contribute to health, such as affordability, access, and housing conditions (Mehdipanah et al., 2019), which impact health through a variety of interrelated mechanisms. Dwellers with few economic resources tend to live in poorer housing conditions and have fewer resources to improve them, contributing to reduced wellbeing, poor health and increased health inequalities (Novoa et al., 2015; Mehdipanah et al., 2019).

To dwell is an essential part of being human (Karjalainen, 1993), inherent to being part of the physical world and inhabiting space. The place of dwelling can be understood as a subsystem of the environment and the main environmental anchor for its inhabitants (Rapaport, 1990). This subsystem enables unique daily activities, “such as eating, sleeping, relaxing, and entertaining family and friends”, and has social-psychological functions related to aspects of autonomy and control, security and retreat, status and identity (Kearns et al., 2011), safety and privacy (Coolen and Meesters, 2012, p. 2) or, as posed by Kearns et al. (2000, p389), “the home as a haven, as a site of autonomy, and providing social status”. Thus, the dwelling encompasses more than -and cannot be reduced to-its material characteristics as a source of shelter. It is also a key element of a meaningful relationship between dwellers and dwelling places (Karjalainen, 1993). However, material housing conditions are relevant for the fulfillment of both the material functions of housing (Orlando et al., 2023) and its social-psychological functions (Kearns et al., 2011). If materially unsuited for its inhabitants’ needs, or of poor quality, the dwelling can become an economic burden, a source of dissatisfaction, and a burden on health and wellbeing.

Both the Universal Declaration of Human Rights in 1948 (article 25.1) and the International Covenant on Economic, Social and Cultural Rights (article 11) (CESCR, 1966), recognized adequate housing as a component of the right to an adequate standard of living. In 1991 the meaning of adequate housing was further developed, describing it as the right to live in security, peace and dignity, and not merely as the shelter provided by a roof or as a simple commodity (CESCR, 1991). Adequate housing implies affordability, accessibility, security of tenure, protection against forced evictions, adequate location, cultural adequacy, and habitability (OHCHR, 2014; UN-Habitat, 2022). Furthermore, ensuring access to adequate, safe and affordable housing, and basic services for all, together with the upgrading of slums, is target 11.1 of the 2030 Agenda for Sustainable Development (UN-Habitat, 2015a). All individuals and families are entitled to adequate housing, without being subject to any form of discrimination (CESCR, 1991).

### 1.2 Habitability and housing satisfaction

Habitability refers to the material dimension of the dwelling or the fulfillment of its material functions. Poor habitability conditions are known risk factors for poor physical and mental health (Mehdipanah et al., 2019). The right to adequate housing explicitly encompasses physical safety, adequate space, protection against cold, dampness, heat, rain, and wind, and other threats to health (CESCR, 1991). More broadly, habitability can be understood as the provision of material conditions that are good enough to live in (Vima-Grau et al., 2021). While different authors’ definitions of habitability vary in their components, they generally include indoor light, acoustic comfort, the arrangement of interior spaces, overcrowding, and the maintenance of installations and functioning of facilities (Corral-Verdugo et al., 2011). A dwelling in poor material conditions will have poor habitability in those components related to the nature of the material deficiencies that affect it. Improved habitability should also reduce stress and situations that deteriorate relationships in the home (Corral-Verdugo, 2010), and lead to improvements in health, thus contributing to residents' overall quality of life (Thomson et al., 2013).

Residential satisfaction, including housing satisfaction, is considered an indicator of wellbeing and a core element in quality of life (Aragonés et al., 2017), as well as a component of life satisfaction, which is positively associated with mental health, health behaviours and some physical health outcomes (Kim et al., 2021). Housing satisfaction refers to the emotional bond or cognitive-affective relation with the dwelling, and to the fulfilment of its material and social-psychological functions, including aesthetic needs (Giuliani, 2004). Housing satisfaction and dissatisfaction have been variously defined. Abidin et al. (2019) define housing satisfaction as “the feeling of contentment when one has or achieves what one needs or desires in a house”. Galster (1987) defines housing dissatisfaction as the gap between the actual and the desired housing situation. According to Gifford (2001), together with the evaluation of the dwelling, satisfaction also involves “purpose and comparison”, such that the perceived and preferred physical qualities of a residence, as well as the gap between them, are predictors of satisfaction.

In this sense, measures of perceived habitability and residential satisfaction are indicators of improved housing quality after regeneration and, as social determinants of health, an indirect indication of the potential for mid or longer-term health improvement. Habitability and housing satisfaction are interrelated. Poor housing conditions and poor habitability can negatively impact the emotional bond between dwellers and the dwelling (Orlando et al., 2023). According to Coolen and Meesters (2012), the positive or negative evaluation of the different aspects of habitability will influence dwellers’ satisfaction with each of those specific aspects, as well as contribute to overall satisfaction with the dwelling (Coolen and Meesters, 2012). Despite the relevance of these factors, social housing projects are frequently constructed without due consideration of the factors determining dweller's housing satisfaction, whether in Chile (Rodríguez and Sugranyes, 2005) or elsewhere (Riazi and Emami, 2018).

### 1.3 Study context

In Chile, the first legislation concerning housing for working class families dates back to 1906 (Greene and Fuentes, 2006). Over time, housing policy has undergone numerous changes in response to changes in the social, political and/or economic environment, the magnitude of the housing deficit and the growth in informal settlements. In the 1980s, under a neoliberal conception of the state, housing policy reforms transferred a major role in social housing construction to the private sector. Using a demand-side subsidy model, this approach lowered the costs of housing and increased the speed of production, achieving a notable reduction in the massive housing deficit the country faced when returning to democracy in 1989 (Greene and Fuentes, 2006). By 2016, there were 1.626 high-rise social housing complexes, a total of 350.880 apartments, especially concentrated in the Metropolitan (56,4%) and the Valparaíso (17,9%) regions, where the municipalities of Puente Alto and Viña del Mar, concentrated the largest proportions of dwellings (4,4% and 5,2%, respectively) (MINVU, 2018).

One of the solutions that proved efficient in large cities were three to four-story housing blocks (called Type C) containing small, low-cost apartments, which became the most recurrent typology in the social housing programs of the 1980s and 1990s (Greene and Fuentes, 2006). These apartments, considered definitive residences, were accessible to families in extreme poverty, but soon proved incapable of adapting to their needs. They are frequently modified and expanded outside legal or safety standards. Eventually, the poor construction standards of these apartments resulted in the physical degradation of dwellings, neighborhoods and communities, leading to the dissatisfaction of their residents (Hidalgo et al., 2017).

The problems with the Chilean solution to the housing deficit became evident early on (Ducci, 1997), as did the critical conditions of habitability and the dissatisfaction of residents. For example, based on a survey with 1800 participants in three metropolitan cities, one study found that up to 64% of families wanted to leave their homes (Rodríguez and Sugranyes, 2005). In contrast to the quantitative deficit of previous decades, this deterioration of the public housing stock is defined as a qualitative deficit, which encompasses deficiencies in infrastructure (mainly drinking water and sewerage), building materials (quality of floors, roofs and walls) and sometimes also legal problems such as irregular tenure (Greene and Fuentes, 2006; ONU-Habitat, 2015). Today, in addition to the urgency of a solution to the housing shortage that has grown in the last years (MINVU, 2022), Chile faces the urgency of the qualitative crisis of the existing social housing stock.

In line with United Nation's New Urban Agenda and international efforts in urban regeneration and dwelling rehabilitation (UN, 2017), MINVU seeks to respond to this qualitative crisis through the *Programa de Regeneración de Conjuntos Habitacionales* (MINVU, 2018), an integral regeneration program which offers dwelling and block renovation (together with providing with information as to the proper use and maintenance of installations), improvements to the built environment and community interventions.

### 1.4 Research question and study objectives

Habitability as a critical condition of housing, and housing satisfaction as the lived experience of such condition, are frequently assessed in qualitative and quantitative studies of social housing in Chile, describing very poor conditions, but no studies have yet assessed the impact of social housing regeneration on these outcomes. Further, studies in Latin America and other middle-income countries have focused on interventions concerning slums and implementation of basic infrastructure but as far as we are aware, there are no documented longitudinal studies that assess the impact of urban regeneration of formal social housing (Baeza et al., 2021; Henson et al., 2020).

The assessment of both material improvements and residents' appraisal of these improvements is relevant because each is related to health-related outcomes, physical and/or mental. And, while several authors note that objective attributes influence peoples’ assessment and satisfaction with those attributes (Marans and Stimson, 2011), they do not necessarily reflect the same housing problems.

In this study, we aim to analyze the impact of urban regeneration on the features of habitability and housing satisfaction subject to intervention, and on overall housing satisfaction in two social housing villas intervened by the *Programa de Regeneración de Conjuntos Habitacionales.* Secondarily, we aim to explore the specificity of those impacts in relation to the characteristics of the intervention, which differ by villa. Third, we aim to assess whether these improvements improve overall housing satisfaction. Our hypothesis is that the intervention (housing renovation) improves reported habitability and reduces dissatisfaction across those features directly improved by the intervention, and –consequently, as noted by Coolen and Meesters (2012), reduces dissatisfaction with the dwelling overall.

## 2 Materials and methods

### 2.1 The RUCAS project

The *Regeneración Urbana, Calidad de Vida y Salud* - RUCAS project is a natural experiment, capitalizing on the urban regeneration program's intervention in two peripheral urban social housing complexes (hereinafter, “villas”, based on local usage) in central Chile. As a natural experiment, the study can robustly evaluate the impact of the above-mentioned program on residents's quality of life and health (Baeza et al., 2021). Habitability and housing satisfaction are key outcomes of the RUCAS study, the former considered a direct intervention effect, and the latter a health-related outcome in the study's conceptual framework (Baeza et al., 2021).

In the RUCAS framework, we propose that the intervention on the material conditions of the dwellings will result in improved habitability and in greater housing satisfaction (or reduced dissatisfaction), considered a health-related outcome (Baeza et al., 2021).

Further details of the RUCAS study and the development of instruments for data collection are described elsewhere (Baeza et al., 2021; Valdebenito et al., 2023). RUCAS is an ancillary study of the *Salud Urbana en América Latina* (SALURBAL) Project (Diez-Roux et al., 2019). The RUCAS study protocol was approved and annually revised by the Health Sciences Scientific Ethics Committee of Pontificia Universidad Católica de Chile with ID #170727004.

## 3 Study cases

One villa, built in 1993, is in Viña del Mar, and the other, built in 1997, in Santiago. Both villas are composed exclusively of three-story “Type C” housing blocks, with apartments of around 40 m^2^, well below current defined standards of space for a typical household of 4 members (Chateau et al., 2020). The villa in Santiago is located in the southernmost limit of the city, in a high-density social housing area with scarce vegetation, which favors the occurrence of heat islands (Romero et al., 2010). The Viña del Mar villa is located in the northern hills of the city, with better conditions for ventilation and lower temperatures (Flamant et al. forthcoming).

## 4 Data and sample

Data comes from survey waves 1 to 5 of the RUCAS study. We use the term “wave” to describe each data collection time point. We collected data biannually, as the intervention unfolded: during or immediately after the warm season in summer (3 waves) and immediately after the cool season in winter (2 waves). At baseline, study participants were recruited following a census strategy among those eligible for intervention. The sampling frame was provided by MINVU in the case of Santiago; in Viña del Mar all households at the time of our baseline measures were eligible households, so we visited all dwellings in the villa (Baeza et al., 2021). Households representing security concerns, and dwellings used for non-residential purposes were excluded. The survey respondents were the homemakers of each household. Surveys were conducted by trained interviewers: face-to-face in waves 1 to 3 and by telephone in waves 4 to 5, during the COVID pandemic. For this study, the sample are the homemakers of all households that participated at baseline (wave 1).

## 5 Study variables

### 5.1 Exposure variable: the intervention

In Viña del Mar the dwelling intervention began in 2017, shortly before baseline measurements. It included expansion of the dwellings from 42 to 57 m^2^ through the attachment of additional 15 m^2^ where the kitchen was relocated, liberating indoor space, together with a laundry and small balcony; utilities (water, electricity, gas and sewage) were renovated and relocated; gas water-heaters were installed; exterior walls were insulated and waterproof painted; and roofs were entirely renovated. The construction process lasted from eight to 12 months. The process is described in greater detail in Chateau et al. (2020), together with a more detailed photographic record of the evolution of the works. Finally, as an integral part of the intervention once it was completed, residents were given guidance on correct user behaviors, such as ventilation, toilet use, and wet laundry management.

In Santiago the dwelling intervention corresponded to the Direct Subsidy for Existing Social Housing (PPPF for its acronym in Spanish), as a component of the broader regeneration program. It was implemented between April 2019 (after baseline measurements) and January 2020. The intervention included full renovation of bathrooms and kitchens; renovation and relocation of utilities (water, electricity, gas and sewage); installation of gas water-heaters with due ventilation; exterior walls were painted but not insulated; roofs were entirely renovated; and roofs and facades were cleared of objects to proceed with the works. Indoor remodeling was tailored to the preferences of each family, with, for example, enlargement of the bathroom or kitchen at the expense of other indoor space or painting of interior walls. The construction work lasted around two weeks per dwelling.

In both villas the works were carried out with the families living inside the dwelling, and advanced progressively across groups of building blocks, according to the master plan established by the regeneration program. This plan responded to constructive or administrative considerations, there was no systematic geographical pattern nor an association with any features relevant to this study.

We created a three-category variable for dwelling intervention status: intervened (I), meaning all the works are finished; under intervention (UI), meaning the works are being carried out; and non-intervened (NI). Intervention status was assessed at every wave and confirmed by the local MINVU.

### 5.2 Outcomes variables

Habitability and satisfaction were assessed in relation to indoor temperature, natural lighting, space, acoustic isolation, mold and leaks in successive survey waves as the intervention advanced. We measure these two types of outcomes to provide more than one form of assessment as to what were thought to be the most critical health-related housing conditions, and to provide both a description of the extent of actual improvement in housing conditions (albeit self-reported), and of the extent to which housing dissatisfaction was reduced, as a more subjective and situated assessment.

**Habitability**: perception of house very cold in winter (“In the winter, is your house very cold?“), of house very hot in summer (“In the summer, is your house very hot?“) and annoying indoor noise from neighbors (“How often do you hear the conversations and annoying noises from the houses that are attached to yours, be it the houses on the sides or the floor above and the floor below?“) were defined as responses of “always” or “almost always” on a 5-point Likert scale. The presence of clean water leakages (rainwater and/or drinking water) and gray water leakages (sewage water and/or dove feces) were defined as responses of “sometimes, almost always or always” on a 5-point Likert scale.

The presence of visible mold on floors, ceilings or walls of bedrooms and the living room was assessed by the interviewer using the RUCAS Intra-domiciliary observation (IDO) tool (Valdebenito et al., 2023). Measurements were performed at waves 1 to 3 and graded from 0 to 3 according to size of the mold stain. The presence of mold was defined as having at least one grade 2 to 3 mold stain in any of the three locations, indicating the presence of considerable mold overgrowth.

**Dissatisfaction:** with indoor temperature (“How satisfied are you with the temperature of your house?“), with acoustic insulation (“How satisfied are you with the acoustic isolation (isolation of noise from the street, from houses or neighbors)?“), with indoor natural light (“How satisfied are you with the natural light that enters your home during the day?“), with dwelling size (“How satisfied are you with the amount of space in your dwelling?“), and with the dwelling overall (“In general, how satisfied are you with your dwelling?“) were defined as responses of “very unsatisfied” or “unsatisfied” on a 5-point Likert scale.

Given the high levels of poor habitability and dissatisfaction, the reduction of which is a central aim of the intervention, we focused our analysis on the reduction of poor habitability and dissatisfaction. However, we also tested results on satisfaction (“satisfied” or “very satisfied”).

See Appendix Table 1 for further detail about what variables were measured in each wave.

**Table 1:** Baseline characteristics of respondents and their households. The RUCAS study.

		Viña del Mar		Santiago	
		n = 238		n = 718	
	Category	n	%	n	%
Sex	Male	65	27.3%	109	15.2%
	Female	173	72.7%	609	84.8%
Age	15–24	15	6.3%	37	5.2%
	25–44	83	34.9%	216	30.1%
	45–59	77	32.4%	331	46.1%
	60 or more	63	26.5%	134	18.7%
Educational level	Low (<8 years)	50	21.0%	206	28.7%
	Medium (8–12 years)	155	65.1%	471	65.6%
	High (>12 years)	33	13.9%	41	5.7%
Dwellers per bedroom	Mean (SD)	1.5 (0.7)		1.5 (0.7)	
	≤1	99	41.6%	31	4.3%
	(1, 2]	116	48.7%	204	28.5%
	(2, 3]	21	8.8%	373	52.0%
	>3	2	0.8%	140	19.5%
Intervention status	NI (Non-intervened)	188	79.0%	718	100.0%
	UI (Under Intervention)	19	8.0%	0	0.0%
	I (Intervened)	31	13.0%	0	0.0%

### 5.3 Covariates

We included the respondent's sex (male, female), age in years, and educational level based on the completed years of formal education (less than 8 years as low level, 8–12 years as medium level and more than 12 years as high level), all measured at baseline. At the household-level, we included dwellers per bedroom, as the ratio of number of dwellers at the time of each survey wave, to the number of rooms used as bedrooms (continuous variable) and seasonality in two categories (summer, winter), both measured in each wave.

## 6 Analysis

Given the panel design with multiple observations per respondent (up to five), we estimated the association between time-varying treatment status and the binary outcomes using Poisson regression models with robust variance and a random intercept at the respondent level. Poisson regression instead of logistic regression was used to estimate prevalence ratios instead of odds ratios, given the high prevalence of the outcomes (Zou, 2004).

We considered intervention effects to be unconfounded given that the intervention unfolds by groups of adjacent housing blocks without systematic differences between dwellings that are and are not intervened (Baeza et al., 2021). Nevertheless, in addition to unadjusted models, we estimated adjusted PRs by including the covariates, which were selected based on a DAG model, to help explain variance in the outcomes and thus increase statistical power to detect associations with the intervention variable.

Given the telephone application of the surveys in waves 4 and 5 due to COVID19 lockdown measures and the highly specific social and familiar context during quarantines, we performed supplementary analysis excluding waves 4 and 5 to examine whether results differ from the ones obtained including all 5 survey waves.

All analyses were performed using StataSE 14 (StataCorp LP, 2015). A confidence level of 95% was used.

## 7 Results

In Viña del Mar, 238 homemakers were recruited at baseline, of which 87.4%, 73.5%, 55.9% and 60.9% participated in waves 2 to 5, respectively. This yielded 114 respondents with data for 5 waves, 36 with data for 4 waves, 30 with data for 3 waves, 37 with data for 2 waves and 21 with data for 1 wave. Of the total of 899 respondent-wave observations, 68.0% were non-intervened, 14.0% were under intervention and 18.0% were intervened.

In Santiago, 718 homemakers were recruited at baseline, of which 90.8%, 83.4%, 60.4%, and 73.8% participated in waves 2 to 5, respectively. This yielded 389 respondents with data for 5 waves, 162 with data for 4 waves, 60 with data for 3 waves, 53 with data for 2 waves and 54 with data from 1 wave. Of the total of 2933 respondent-wave observations 94.2% were non-intervened, 0.5% were under intervention and 5.3% were intervened.

Table 1 shows the distribution of covariates at baseline (summer season) in each villa. Sociodemographics are similar: over 70% are women, over 60% are aged between 25 and 59 years, and 65% have between 8 and 12 years of schooling. Compared to Viña del Mar, the sample in Santiago has a higher proportion of female (84.8% vs 72.7%), a lower proportion over 60 (18.7% vs 26.5%), a lower proportion of respondents with higher education (5.7% vs 13.9%), and a higher proportion of households in dwellings with more than 3 dwellers per bedroom (19.5% vs 0.8%). At baseline, in Santiago, all the dwellings were non-intervened, while in Viña del Mar, 13.0% had already been intervened.

Table 2 shows the distribution of the outcomes at baseline. Reported poor housing conditions are generally high, but markedly higher in Santiago than in Viña del Mar. The most frequently reported are annoying indoor noise from neighbors (57.1% and 82.0% in Viña del Mar and Santiago, respectively), followed by home perceived very cold in winter (46.3% and 71.8%), presence of gray water leakages (44.1% and 66.3%) and home perceived very hot in summer (33.3% vs 64.8%). Regarding dissatisfaction with the dwelling, one out of four (25.6%) participants in Viña del Mar and up to 45.8% in Santiago reported being dissatisfied with the dwelling overall. The greatest dissatisfaction with specific features of the household concerned acoustic insulation, reaching 62.0% and 81.5% (Viña del Mar and Santiago, respectively), followed by dwelling size (41.6% and 62.3%) and indoor temperature (22.4% and 50.6%). The least dissatisfying feature was natural indoor light (18.6% and 26.6%).

**Table 2:** Baseline prevalence of poor habitability conditions and dissatisfaction. The RUCAS study.

	Viña del Mar		Santiago	
	n = 238		n = 718	
	n	%	n	%
**Poor habitability**				
Perception of house very cold in winter	105	46.3%	507	71.8%
Perception of house very hot in summer	79	33.3%	465	64.8%
Perception of annoying indoor noise from neighbors	136	57.1%	587	82.0%
Presence of clean water leakages	59	24.9%	348	48.5%
Presence of gray water leakages	105	44.1%	476	66.3%
Presence of visible mold in bedrooms	54	22.8%	207	29.0%
Presence of visible mold in living room	19	8.0%	97	13.6%
**Dissatisfaction**				
Dissatisfaction with indoor temperature	53	22.4%	363	50.6%
Dissatisfaction with acoustic insulation	147	62.0%	584	81.5%
Dissatisfaction with indoor natural light	44	18.6%	191	26.6%
Dissatisfaction with dwelling size	99	41.6%	447	62.3%
Dissatisfaction with the dwelling overall	61	25.6%	329	45.8%

Table 3 describes the proportion of respondents in non-intervened (NI), under intervention (UI) and intervened (I) dwellings across survey waves. In the Viña del Mar sample, the proportion of intervened dwellings was largest in wave 2 (20.7%; n = 43), given losses to follow-up in subsequent survey waves. In Santiago, the proportion of intervened dwellings is limited to around 8% of the households, which received the intervention between waves 2 and 3, and only a small number were surveyed while they were undergoing the intervention.

**Table 3:** Proportion of respondent-wave observations according to their intervention status across survey waves. The RUCAS study.

		Survey wave									
		Wave 1		Wave 2		Wave 3		Wave 4		Wave 5	
		(Apr - 2018)		(Aug −2018)		(Mar - 2019)		(Aug −2020)		(Mar - 2021)	
		n	%	n	%	n	%	n	%	n	%
Viña del Mar (N = 899)	NI	188	79.0%	165	79.3%	139	79.4%	73	54.9%	46	31.7%
	UI	19	8.0%	0	0.0%	0	0.0%	38	28.6%	69	47.6%
	I	31	13.0%	43	20.7%	36	20.6%	22	16.5%	30	20.7%
	Total	238	100%	208	100%	175	100%	133	100%	145	100%
		Wave 1		Wave 2		Wave 3		Wave 4		Wave 5	
		(Jan - 2019)		(Jul −2019)		(Mar - 2019)		(Aug −2020)		(Jan - 2021)	
		n	%	n	%	n	%	n	%	n	%
Santiago (N = 2933)	NI	718	100.0%	616	94.5%	550	91.8%	398	91.7%	482	90.9%
	UI	0	0.0%	15	2.3%	0	0.0%	0	0.0%	0	0.0%
	I	0	0.0%	21	3.2%	49	8.2%	36	8.3%	48	9.1%
	Total	718	100%	652	100%	599	100%	433	100%	530	100%

NI: non-intervened; UI: under intervention; I: intervened.

Table 4 shows prevalence ratios of perceived habitability and dissatisfaction associated with the intervention status (adjusted for covariates) and their corresponding 95% confidence intervals. Adjusted results do not greatly differ from unadjusted results (See Appendix Table 2).

**Table 4:** Prevalence ratios (PR) with 95% confidence interval of poor habitability conditions and of dissatisfaction associated with intervention status, adjusted for sex, age and SES of the respondent, dwellers per bedroom, and season. The RUCAS study.

			Viña del Mar		Santiago	
			PR	CI 95%	PR	CI 95%
Habitability	Perception of house very cold in winter	NI	1		1	
		UI	0.79	0.63–0.99	0.80	0.56–1.15
		I	0.48	0.33–0.69	0.94	0.82–1.08
	Perception of house very hot in summer	NI	1.00		1.00	
		UI	0.89	0.63–1.25	0.85	0.53–1.35
		I	0.93	0.65–1.34	1.00	0.87–1.16
	Perception of annoying indoor noise from neighbors	NI	1		1	
		UI	0.81	0.66–1.00	0.75	0.51–1.11
		I	0.63	0.46–0.86	0.97	0.87–1.09
	Presence of clean water leakages	NI	1.00		1.00	
		UI	0.49	0.27–0.92	0.64	0.27–1.51
		I	0.24	0.12–0.47	0.24	0.14–0.40
	Presence of gray-water leakages	NI	1		1	
		UI	0.82	0.59–1.12	0.78	0.45–1.36
		I	0.33	0.21–0.51	0.23	0.14–0.36
	Presence of visible mold in bedrooms	NI	1.00		1.00	
		UI	0.86	0.36–2.06	0.36	0.10–1.27
		I	0.31	0.15–0.64	0.60	0.35–1.01
	Presence of visible mold in living room	NI	1		1	
		UI	0.78	0.47–1.29	0.00	0.00–0.00
		I	0.39	0.25–0.60	0.10	0.01–0.71
Dissatisfaction	Dissatisfaction with indoor temperature	NI	1.00		1.00	
		UI	1.11	0.83–1.48	0.68	0.38–1.23
		I	0.52	0.32–0.82	0.93	0.77–1.13
	Dissatisfaction with acoustic insulation	NI	1		1	
		UI	0.81	0.69–0.96	0.81	0.58–1.13
		I	0.55	0.41–0.72	0.94	0.85–1.05
	Dissatisfaction with natural indoor daylight	NI	1.00		1.00	
		UI	2.33	1.60–3.40	1.12	0.51–2.46
		I	0.66	0.36–1.24	0.74	0.46–1.18
	Dissatisfaction with dwelling size	NI	1		1	
		UI	0.67	0.50–0.88	0.89	0.60–1.32
		I	0.13	0.06–0.28	0.81	0.63–1.05
	Dissatisfaction with dwelling overall	NI	1.00		1.00	
		UI	0.74	0.48–1.13	0.52	0.24–1.12
		I	0.43	0.25–0.73	0.45	0.31–0.65

NI: non-intervened; UI: under intervention; I: intervened.

In Viña del Mar, analysis of habitability reports showed that the perception that the house is too cold in winter was 52% lower in intervened compared to not intervened households, whereas no significant differences are observed for reports of the house being too hot in summer. The report of annoying indoor noise is reduced by 37%. The presence of leakage of clean water decreased by 76%, and gray water leakage decreased by 67%. The presence of mold in bedrooms decreased by 69% and 61% in living rooms. Dwellings under intervention show reductions in perceived indoor cold, annoying indoor noise, and leakage of clean water (21%, 19% and 51% reductions, respectively).

We also observed that the intervention was associated with significantly lower prevalence rates of all dissatisfaction measures, except indoor natural light. Compared to non-intervened dwellings, in intervened dwellings the prevalence of dissatisfaction was 48% lower for indoor temperature, 45% lower for acoustic insulation, 87% for dwelling size, and 57% for the dwelling overall. Dwellings under intervention show significantly lower dissatisfaction with acoustic insulation (19%) and dwelling size (33%), and a notably and statistically significant higher prevalence in dissatisfaction with indoor natural light (233%).

In Santiago, we observed improvements in the presence of water leakages, both clean (76% lower prevalence) and gray (77% lower prevalence), as well as reductions in the presence of mold in living rooms (90% lower prevalence) and bedrooms (40% lower prevalence, close to statistical significance). Although dissatisfaction with all measured items was slightly lower among intervened dwellings, only the prevalence of overall dissatisfaction was significantly lower (55% lower). Dissatisfaction with indoor natural light (0.74; 0.46–1.18) and with dwelling size (0.81; 0.63–1.05) were also reduced in intervened households, although confidence intervals overlapped 1.

We performed sensitivity analyses using satisfaction as our outcome variable, observing a general consistency of results (See Appendix Tables 2a and 2b). We also assessed the potential impact of the COVID19 pandemic, prolonged lockdown, and telephone survey applications on our results, repeating our analysis excluding the COVID period (waves 4 and 5) and found roughly the same results, except for the lack of statistical significance of some associations given more limited statistical power (See Appendix Table 3a y 3 b).

## 8 Discussion

To our knowledge, this is the first longitudinal study to analyze the impact of housing renovation among poor urban dwellers of formal social housing in Latin America. A unique feature of this study in the Latin American context is the assessment of a housing renovation intervention of formal and definitive residences, which contrasts with most available literature in the region, which focuses on the upgrading of slums (Henson et al., 2020). Our key findings are, first, that dwelling renovation can improve reported habitability conditions and significantly reduce housing dissatisfaction in critically deteriorated, substandard dwellings among urban-poor homemakers in formal social housing. In addition, the analysis of two cases with different interventions allowed us to test and provide evidence of the specificity of this impact: reported habitability and housing satisfaction improved for those features of the dwelling that were improved by the intervention. Finally, we find that, in both cases, the intervention led to equivalent improvements in overall housing satisfaction.

### 8.1 Baseline situation

Housing is a key social determinant of health, influenced by structural determinants such as social, macroeconomic, and public policies, with the burden of substandard housing falling disproportionately on low-income, vulnerable populations (Mwoka et al., 2021), where primary research is deemed especially necessary (Vima-Grau et al., 2021). In line with this, we contribute with evidence on the scope and extent of inadequate and substandard housing in peripheralized social housing complexes in central Chile.

In line with previous studies (Rodríguez and Sugranyes, 2005), both habitability and dissatisfaction were critically deficient at baseline, responding to the selection criteria for the regeneration program, which is aimed at high-rise social housing complexes in critically deteriorated conditions. Despite differences in magnitude, the most prevalent features of deficient habitability at baseline were the same in both villas, reflecting their shared material and spatial qualities. These features (annoying indoor noise, perceived indoor cold in winter, presence of gray water leaks, and excess indoor heat) are all related to physical and mental health (WHO, 1989). Instead, the main sources of dissatisfaction were not entirely the same in both villas. In this same vein, previous studies in Chile have observed that objective assessments of dwelling habitability do not provide the same results as the assessment of residents’ satisfaction with it (Fadda and Jirón, 2001). Objective measures of environmental attributes have long been considered insufficient to address quality of life, given that the interaction between people and the environment is key to that assessment (Aragonés et al., 2017).

### 8.2 Housing renovation and improvements in habitability

The differences in the breadth and scope of the intervention in the two villas, which had identical original building structures, allowed us to examine the specificity of the impact of renovation on different aspects of habitability and housing satisfaction. Statistically significant improvements concerning precisely those features of the dwellings that were renovated confirm the specificity of the experience. In addition, this contributes with information on the validity of the items in the questionnaire.

In Viña del Mar, the perception of indoor cold in winter, annoying noise from neighbors, leakages, and the presence of mold in bedrooms were all significantly reduced, responding to two of the main aims of the intervention in that villa, which were to provide buildings with better insulation and sanitary installations.

In Santiago, large improvements were observed relative to leaks of clean and gray waters (over 70% reduction in both) and the presence of mold in bedrooms and living rooms. These improvements are related to the renovation of roofs, sanitary installations and the wet areas of the home (kitchen and bathrooms). On the other hand, excessive indoor heat in summer did not improve substantially in Viña del Mar, partly because the intervention did not specifically target indoor heat and partly because of the lower initial frequency of problem (33%) and the milder summer temperatures. In Santiago, where the problem of excess heat was very prevalent, no improvements were observed relative to temperature (or noise), consistent with the fact that the renovations did not include insulation of the buildings.

### 8.3 Reduction of dissatisfaction

We assess satisfaction in several aspects of habitability, an approach that contributes to a better understanding of the complex factors involved in dwelling satisfaction (Clapham et al., 2018). As with habitability, our results showed a positive impact of the intervention on satisfaction with those features of the dwellings that were improved by the intervention.

In Viña del Mar, the large reduction in dissatisfaction with dwelling size is especially health-relevant since insufficient space affects privacy and autonomy, as well as personal and family life (Orlando et al., 2023; Carrasco et al., 2021). And while the perception of lack of space is not an absolute fact, but related to the needs of households (Karjalainen, 1993), the size of social housing solutions is, for the most part, unsuited to the needs of low-income families who tend to include a relatively large number of people (Rodríguez and Sugranyes, 2005), making this a key issue in regeneration policy. The reduction in dissatisfaction with space responds to the 40% increase in dwelling size, although it may not necessarily have reached full satisfaction of family needs. We cannot, however, determine to what extent the reorganization of indoor space (the kitchen moved to the expanded area and, consequently, other indoor spaces were enlarged or modified) contributed to this notable reduction in dissatisfaction above and beyond the actual increase in size.

In fact, in Santiago, dwellings were not expanded, but we do observe a small reduction in dissatisfaction with dwelling size, which would be related to the tailored reorganization of indoor space, which included the enlargement of the bathroom or kitchen (or none) according to the resident's preferences and needs, better accommodating dwelling functions and enabling activities that lead to higher well-being (Orlando et al., 2023; Foye, 2017). The implication is that indoor space is largely and mainly related to actual size, but that the tailored reorganization of indoor space can also contribute to improved housing satisfaction given a better fit between space and family needs. A Cochrane review found that to promote good health, housing should be appropriately sized for homeowners and their needs (Thomson et al., 2013). Insufficient dwelling space is related to overcrowding, which is in turn associated with increased health problems related to infectious disease, mental health, lack of privacy and domestic violence (Carrasco et al., 2021; WHO, 2011)

Also, interventions appear to have benefits beyond their primary objective (Shaw, 2004). Dissatisfaction with natural indoor light was somewhat reduced in both villas, despite this not being a primary objective of the intervention. This may be partly explained because the prevalence of dissatisfaction with indoor light at baseline was low, and hence, there is limited space for improvement. In addition, in Viña del Mar dwellings were expanded at the expense of indoor light in other rooms of the apartment. However, the intervention included the construction of new luminous kitchens, providing a marked increase in natural indoor light, which was highly appreciated by participants in qualitative interviews (Orlando et al., 2023). In Santiago, the observed improvement in natural indoor light may be due to the clearing of roofs and facades of objects that obstructed the entry of natural light, and to the new white wall tiling of bathrooms and kitchens, and the painting of indoor walls, increase the ability of walls to reflect the light coming in through the windows, thus improving indoor luminosity conditions.

Finally, as hypothesized, overall housing dissatisfaction was also reduced, and to a large extent. This reduction was roughly equivalent in both villas, despite the differences in the nature and magnitude of the intervention. This supports the notion that material housing interventions, despite addressing specific habitability dimensions, have the potential to improve satisfaction overall, and that a general measure of satisfaction is similarly sensible to improvements of different characteristics and intensities. For example, a larger bathroom can improve the safety and comfort with which an adult looks after a child's hygiene (Carrasco et al., 2021), and renovated kitchens can improve the experience of cooking and associated tasks. Given respondents are homemakers, and mainly women (female head of household or *dueña de casa*), who are mostly in charge of these types of chores, there are greater chances that these improvements will improve their overall housing satisfaction. The results from the Santiago villa suggest that significant effects on housing satisfaction can be obtained if a more limited intervention is tailored to the households' needs. This implies they may be a good alternative to major interventions that, although provide a more comprehensive solution, take more time and resources to implement. This potential to improve overall housing satisfaction is a key result, considering that housing satisfaction is a component of quality of life (Turkoglu et al., 2006) and associated with mental and general health (Bjørndal et al., 2024; Knöchelmann et al., 2020).

### 8.4 Dwellings under intervention

Most of the intervention is carried out with the dwellers residing inside the apartment, substantially affecting their living conditions. We note that, although smaller in magnitude, results observed for dwellings undergoing intervention in Viña del Mar were similar. These dwellings were, for the most part, already insulated and expanded, but the expansion was not yet in use. In these we observed significant reductions in dissatisfaction with acoustic insulation and dwelling size. We also observe a very large and statistically significant increase in dissatisfaction with indoor natural light. This was to be expected given that the buildings were covered with dark mesh during the intervention, substantially reducing the amount of natural light entering the apartments (See Appendix Fig. 1). Finally, overall dissatisfaction with the dwelling only improved only after the intervention was completed, possibly due to the inconveniences of the works and the uncertainty regarding the outcome.

### 8.5 Study limitations

The main study limitation is the small number of dwellings intervened in Santiago, which makes it difficult to observe statistically significant results, despite which we observe results that are consistent with the intervention conducted. Another limitation is that most of our data on habitability conditions are self-reported. Nevertheless, the observed improvements on both subjective and objective (interviewer assessment of mold) measures of habitability are consistent with the interventions performed.

Another potential limitation of this study is that our appraisal combines different lengths of exposure to the intervention, ranging from 6 months to 4 years in the Viña del Mar villa, in accordance with the nature of the study's step-wedged design. Some studies have observed a decrease in satisfaction before the intervention (e.g., moving houses), which would increase effect sizes; others have suggested that the positive effect of a change in habitability on housing satisfaction may be reduced over time, after a period of adaptation to the new conditions, although evidence is not conclusive (Clapham et al., 2018). The evaluation of these time trends was not the focus of this study, but the assessment of this potential downward trend in housing satisfaction over time appears as a policy-relevant issue to be further explored. However, if the material conditions of renovated houses are of good quality and durability, the pre-intervention levels of dissatisfaction should not be reached. On the other hand, dissatisfaction could be expected to rise, maybe even higher than baseline, if the expectations that the dwelling would improve are frustrated.

Being homemakers, our subjects in this study are predominantly women. Hence, results are more representative of the experiences and perceptions of women than men, which are not necessarily the same. For example, a study in the UK found that an increase in living space (rooms per person) had only a weak effect on the life satisfaction and mental health of men (Foye, 2017). Despite this, we consider the selection of homemakers to be a strength of our study, since women spend more time in the home and generally deal more closely with its problems. Women homemakers are thus in better condition to report about the conditions of the dwelling and the household and are also among the most affected by its conditions (Pevalin et al., 2008), making their experiences and perceptions especially relevant.

### 8.6 Policy implications

The Chilean solution to the housing crisis implemented in the 1990s has been replicated in other countries in the region, which are showing signs of reproducing the qualitative housing crisis as well (UN-HABITAT, 2015b). In Chile, diverse “second generation” programs have attempted to address this problem, ranging from specific housing, or built environment renovation initiatives, the demolition of housing blocks, to more comprehensive policies such as the *Programa de Regeneración de Conjuntos Habitacionales* (Fuentes et al., 2021; Ministerio de Vivienda y Urbanismo MINVU, 2018). In this sense, Chile has pioneered solutions and accumulated an experience that can be useful for other countries in Latin America, a region where the qualitative housing deficit has been estimated to affect over 30% of urban households (Bouillon, 2012). The housing regeneration solution, however, has proven to be complex and slow to implement, especially when more complex interventions designs are in place (Chateau et al., 2020).

In this line, a first lesson from this and prior studies must be that the answer to the quantitative housing emergency, cannot overlook housing quality. This is especially policy relevant considering the extent to which the quantitative housing problem has become a pressing emergency, in Chile and abroad. The very poor habitability conditions of these dwellings and the high levels of dissatisfaction of their residents should be considered unacceptable to public policy aiming to solve the housing crisis, and as a failure to meet the human right to *adequate* housing. The dwelling is called to be the home, the haven from which to go out and explore the world, and to which come back from (Heller, 1987). If governments are to grant their populations sustainable housing solutions, quantity and quality must go hand in hand. Such considerations also apply to the built environment, as well as service provision.

Second, the key lesson from this study is that housing renovation appears as a promising intervention for the qualitative housing crisis. Through the improvement of the material conditions of the dwelling, habitability improves, and with it, housing satisfaction. The material adequacy of the dwelling enables its many material, social and psychosocial functions, improving the lived experience of the dwellers and their general sense of wellbeing (Orlando et al., 2023). Improved habitability (and satisfaction) can also lead to improvements in physical and mental health, contributing to the residents' overall quality of life (Thomson et al., 2013). In this sense, residential regeneration has been considered a public health intervention on a key social determinant of health (Shaw, 2004; Bond et al., 2013). Habitability and satisfaction improvements are both specific to the interventions in place, but also contribute to overall housing satisfaction, an important component of quality of life (Clapham et al., 2018).

Third, it is of note that two interventions of different breath and scope can equally improve overall housing satisfaction. On the one hand, more limited interventions may enhance housing satisfaction if it is tailored to the family's needs, allowing for a more functional reorganization of space and its uses. This, however, merits further research. On the other hand, we find that specific material improvements have the potential to produce both direct and indirect benefits on habitability (Shaw, 2004). For example, improving rooftops and facades to prevent water leakages requires clearing rooftops and walls, secondarily increasing the entry of natural daylight into the homes. In a previous qualitative study, we observed how increased indoor space improved the ability of dwellers to clean their houses more thoroughly, and how renovation encouraged dwellers to make further improvements in the dwellings (Orlando et al., 2023). On the other, and in a similar fashion, specific material improvements of the dwelling can benefit health more broadly. For example, thermal insulation can provide more affordable thermal comfort, reducing the need for heating and thus air pollution, all of which, in turn, may be relevant for respiratory health (Howden-Chapman et al., 2007). A more efficient energy consumption may allow homemakers to heat more rooms and increase the amount of useable space (Thomson et al., 2013), which may lead to a more extended use of the dwelling, allowing increased levels of privacy, and helping with relationships within the home (Thomson et al., 2013) potentially also reducing domestic violence by reducing stress and situations that deteriorate relationships in the home (Corral-Verdugo et al., 2011).

So, while dissatisfaction with specific dwelling features was significantly more reduced with a more comprehensive intervention that included expansion and new functional spaces, it is valuable to note that if such solutions are not feasible, a more limited material renovation of the dwelling can still be a necessary and potentially significant intervention. While it remains to be determined which specific interventions should be prioritized, we find that improving the material conditions and basic functioning of the dwelling, while setting other priorities according to families' needs and preferences, has significant potential to improve habitability and housing satisfaction. Today, the pressure of climate change and increased heat, superimposed on precarious habitability conditions, can significantly affect the urban poor's wellbeing and health (Palmeiro-Silva et al., 2020). This is critical for Central Chile, where warmer and drier summers are expected during this century, as has been observed in the last decade (Garreaud et al., 2017). For the urban poor, heat adds to the problems of energy poverty they face in dealing with the cold in winter. It is thus imperative that therenovation of urban social housing moves forward at a faster pace to address the additional risks that global warming imposes on lower-income families living in substandard housing.

## 9 Conclusions

Renovation of formal social housing in a state of advanced deterioration can improve habitability and significantly reduce housing dissatisfaction among urban-poor homemakers in formal social housing. And while improvements in both reported habitability and housing satisfaction were specific to the interventions in place, both led to significant improvements in overall housing satisfaction. This study highlights two issues in housing policy towards achieving the human right to adequate housing for all: the critical need to build quality social housing, and the benefits and urgency of housing renovation for the urban poor.

## EA

The RUCAS study protocol was approved and annually revised by the Health Sciences Scientific Ethics Committee of Pontificia Universidad Católica de Chile with ID #170727004.

## CRediT authorship contribution statement

**F. González:** Writing – review & editing, Writing – original draft, Visualization, Validation, Software, Project administration, Methodology, Investigation, Funding acquisition, Formal analysis, Data curation, Conceptualization. **F. Baeza:** Writing – review & editing, Visualization, Validation, Project administration, Investigation, Funding acquisition, Conceptualization. **R. Valdebenito:** Writing – review & editing, Project administration, Investigation. **B.N. Sánchez:** Writing – review & editing, Methodology. **A. Diez-Roux:** Writing – review & editing, Writing – original draft, Visualization, Validation, Supervision, Resources, Methodology, Investigation, Funding acquisition, Conceptualization. **A. Vives:** Writing – original draft, Visualization, Validation, Supervision, Resources, Methodology, Investigation, Funding acquisition, Conceptualization.

## Data Availability

The data that has been used is confidential.
