# Supplementary material for: Improvements in habitability and housing satisfaction after dwelling regeneration in social housing complexes. The RUCAS study
Source: Soc Sci Med. 2024 Aug;355:117090. doi: 10.1016/j.socscimed.2024.117090 (PMC11364156; doi:10.1016/j.socscimed.2024.117090)
Supplement: Multimedia component 1 [file mmc1.docx]

## APPENDIX

Table 1. Wave measurement of each variable. The RUCAS Study.

Table 2. Prevalence ratio (PR) with 95% confidence interval for satisfaction, crude and adjusted for sex, age and SES of the respondent, persons per bedroom, and season for (a) Viña del Mar, and (b) Santiago. The RUCAS study.

1. Viña del Mar

1. Santiago

Table 3. Prevalence ratio (PR) with 95% confidence interval for poor habitability conditions and dissatisfaction, adjusted for sex, age and SES of the respondent, persons per bedroom, and season considering data from waves 1 to 3, and waves 1 to 5 for (a) Viña del Mar, and (b) Santiago. The RUCAS study.

1. Viña del Mar

1. Santiago

Figure 1. Phases of the intervention in the Viña del Mar *villa:* a. non-intervened; b. under intervention; and c. intervened. The RUCAS Study.

a. b. c.


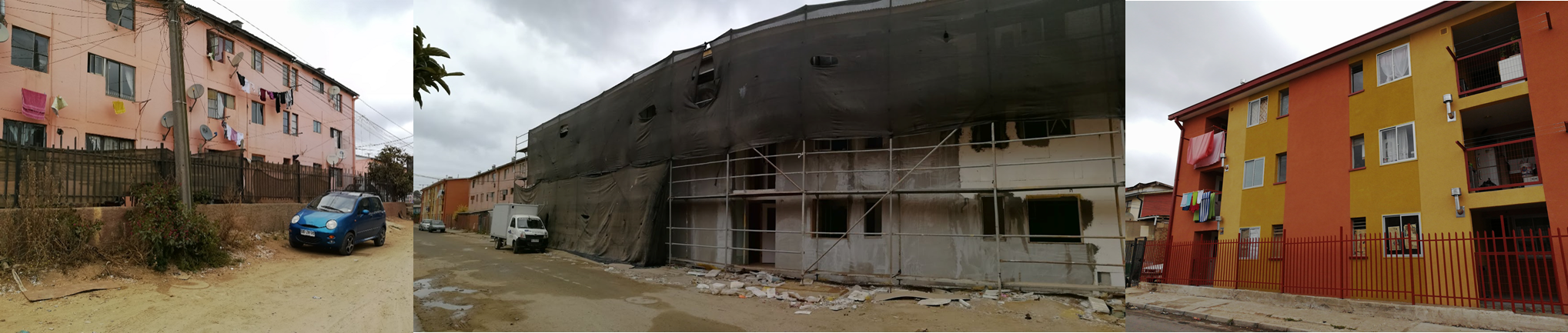


Source: RUCAS collection.
